# Association between breastfeeding, parents’ body mass index and birth weight with obesity indicators in children

**DOI:** 10.1186/s12887-022-03641-3

**Published:** 2022-10-18

**Authors:** Maurício dos Santos, Gerson Ferrari, Clemens Drenowatz, José Matheus Estivaleti, Eduardo Rossato de Victo, Luis Carlos de Oliveira, Victor Matsudo

**Affiliations:** 1 Centro de Estudos do Laboratório de Aptidão Física de São Caetano do Sul (CELAFISCS), São Caetano do Sul, Brazil; 2 Escuela de Ciencias de la Actividad Física, el Deporte y la Salud, Universidad de Santiago de Chile, USACH, Santiago, Chile; 3 Facultad de Ciencias de la Salud , Universidad Autónoma de Chile, Santiago, Chile; 4 Division of Sport, Physical Activity and Health, University of Education Upper Austria, 4020 Linz, Austria; 5 Departamento de Pediatria, Escola Paulista de Medicina (EPM), Universidade Federal de São Paulo (UNIFESP), São Paulo, Brazil

**Keywords:** Epidemiology, Breastfeeding, Obesity, Motor Activity

## Abstract

**Background:**

Childhood obesity is potentially affected by breastfeeding, parents’ body mass index and birth weight. Thus, this study aimed to verify the association between breastfeeding, parents’ body mass index and birth weight with obesity indicators in children.

**Methods:**

This is a cross-sectional study, including data from 402 schoolchildren between 9 and 11 of age in the city of São Caetano do Sul, Brazil. Parents or guardians answered a questionnaire about breastfeeding (month), birth weight (kg), and parental body weight and height (parents’ body mass index [kg/m^2^] was calculated). Body mass index (kg/m^2^), waist circumference (cm) and body fat (%), determined via bio-impedance, were measured and used as obesity indicators. Multi-level linear regression models were used to assess the respective associations adjusted for the potential confounders.

**Results:**

Considering body mass index of children, the overall prevalence of eutrophic, overweight and obese were 58.2%, 20.9% and 17.2%, respectively. Significant and positive correlations were observed between breastfeeding, maternal as well as paternal body mass index and the children’s body mass index, body fat and waist circumference. Birth weight was weakly and positively associated with body mass index and body fat but was not associated with waist circumference. After adjusting for school, sex, age, race/ethnicity, annual household income, sedentary time and moderate-to-vigorous physical activity, maternal body mass index and birth weight were positively associated with children’s body mass index (β: 0.228; 95%CI: 0.142; 0.314 and β: 0.001; 95%CI: 0.001; 0.002), body fat (β: 0.484; 95%CI: 0.297; 0.671 and β: 0.002; 95%CI: 0.001; 0.003) and waist circumference (β: 0.509; 95%CI: 0.304; 0.715 and β: 0.003; 95%CI: 0.001; 0.005). Breastfeeding was not associated with any obesity indicators.

**Conclusion:**

Maternal body mass index and birth weight were associated with children’s obesity indicators. The perinatal environment, therefore, appears to be a critical contributor to childhood obesity and public policies need to address parental obesity in order to tackle childhood obesity.

**Trial registration:**

The International Study of Childhood Obesity, Lifestyle and the Environment (ISCOLE) is registered at (Identifier NCT01722500).

## Background

Obesity has been identified as one of the major threats to future public health [1]. Of particular concern is the dramatic increase in childhood obesity, which has been observed in recent years, not only in high-income countries, but also in low- and middle-income countries [2]. In fact, a recent review reported that the prevalence of childhood overweight and obesity increased by more than 45% between 1980 and 2013 worldwide [3]. Excess body weight during childhood is a strong predictor of adult obesity [4] and other health consequences such as type 2 diabetes and cardiovascular disease in adolescence and adulthood [5]. Thus, it is important to combat obesity and identify the risk factors associated with it.

The benefits of breastfeeding in early childhood are well established in the literature [6]. Breastfeeding is the recommended form of nutrition for the first few months of a children´s life. Data from a meta-analysis showed that breastfeeding was associated with a significant reduction in the risk for childhood obesity [6]. Other studies, however, reported only weak or no association between breastfeeding and childhood obesity [7, 8]. The inconsistent results suggest that the association between breastfeeding and children’s weight status may be moderated by several other variables [9].

Obesity is a multifactorial disorder influenced by genetic, socioeconomic and lifestyle factors [10]. Parental body mass index (BMI), children birth weight, and breastfeeding duration are factors that have been shown to increase the risk of childhood obesity [9–11]. However, lifestyle indicators, such as moderate-to-vigorous physical activity, sedentary behavior may confound the association between breastfeeding, parents’ BMI and birth weight with the risk of later childhood obesity [9, 10, 12]. Thus, the aim of this study was to verify the association between breastfeeding, parental BMI and birth weight with obesity indicators in children.

## Methods

### Study design and sample

The study was carried out in São Caetano do Sul, state of São Paulo, as part of the International Study of Childhood Obesity, Lifestyle and the Environment (ISCOLE) which includes 12 countries, with data from children between 9 and 11 years of age. Details of the ISCOLE study protocol have been previously published [13]. The municipality of São Caetano do Sul, located in the state of São Paulo, Brazil, has an area of 15.3 km^2^ and is located in a subtropical climate [10, 14]. The population of 10-year-old children in the municipality in 2013 was 1,557 (52.1% boys). The municipality has a service economy and the best Human Development Index in Brazil [14, 15].

The study protocol was introduced to school members and parents. After the respective permissions, it was applied in all selected schools. All children aged between 9 and 11 years old and in the 5th grade of elementary school were eligible to participate in the project. All schools were inserted in random order within a list and were selected according to a random draw in each stratum, considering a proportion of 80% (public) to 20% (private). Sixteen public and four private schools were selected in order to obtain a total sample of 500 children (50% of each sex) with 25–30 children from each school. Data collection was carried out between March 2012 and April 2013 and all assessments were carried out during a full week per school. Children were eligible to participate in the study if they: (a) were 9–11 years of age; (b) regularly enrolled in a school in the Sao Caetano do Sul system; and (c) did not have clinical or functional limitations preventing daily physical activity. Details of the sample size calculation and inclusion and exclusion criteria have been previously published [13, 14].

This study was conducted according to the guidelines laid down in the Declaration of Helsinki and all procedures involving human subjects/patients and must have been approved by the Pennington Biomedical Research Center Institutional Review Board and Ethics Committee of the Federal University of São Paulo (number: 332.529). Informed consent was obtained from parents/legal guardian(s) of the children.

A total sample of 584 (297 girls) children was invited to participate in the study and met the inclusion criteria [13, 14]. Participants with missing data (n = 176) were excluded. Thus, the final sample included 402 children (202 girls) with complete data. Details have been published elsewhere [13, 14].

### Breastfeeding, parents’ body mass index and birth weight

A family health history questionnaire was completed by the parents or legal guardians of children between 9 and 11 years of age [13, 14]. The questionnaire contained information on breastfeeding, parental weight and height, and children’s birth weight. Breastfeeding (month) was assessed by the age at which the child completely stopped being breastfed and was analyzed continuously. Data on parental height and weight were collected via questionnaire and consequently the father’s and mother’s body mass index was calculated (kg/m^2^) and analyzed separately. Children’s birth weight (kg) was reported by parents or guardians and was analyzed continuously. Further details on the questionnaire can be found in a previous studies [13, 14].

### Obesity indicators

The variables analyzed were: height, body weight, percentage of body fat (%BF) and waist circumference (WC) [13]. Height was measured to the nearest 0.1 cm using a portable Seca 213 stadiometer (Hamburg, Germany). Body weight and %BF were measured using a Tanita SC-240 scale, portable body composition analyzer (Arlington Heights, IL) after children removed heavy items from their pockets, shoes and socks [16]. Two measurements were obtained and the mean was used in the analysis (the third measurement was obtained if the first two measurements had a difference greater than 0.5 kg or 2.0% of distance for body mass). BMI was calculated from height and body weight (kg/m^2^), and subsequently converted to z-scores based on the World Health Organization (WHO) growth reference data. The nutritional status was classified as: underweight: <-2SD; eutrophic: -2 SD to 1 SD; overweight > + 1 SD to 2 SD; and obese: >+2 SD [17].

WC measurements were made on exposed skin at the end of normal expiration using a non-elastic anthropometric tape midway between the lower margin of the last rib and the iliac crest [13].

### Sedentary time and moderate-to-vigorous physical activity

The Actigraph GT3X accelerometer (ActiGraph, Ft. Walton Beach, USA) was used to objectively monitor sedentary time and moderate-to-vigorous physical activity. The instrument was worn at the waist using an elastic belt, in the mid axillary line on the right side. Participants were encouraged to use the accelerometer 24 h/day for at least 7 days, including two weekend days. Children were instructed to remove the accelerometer only for water activities. To increase compliance, study staff instructed children on how to wear the accelerometer during the initial in-school assessment, and conducted an in-person compliance check 2–4 days after initialization. Further, participants were contacted twice during data collection via telephone (one weekday evening and one weekday) to ensure they were wearing the device, and to address any questions. Accelerometer monitoring procedures were identical to those described in previous studies [13, 18].

The minimum amount of accelerometer data that was considered acceptable for analysis was four days (including at least one weekend day), with at least 10 h/day of wear-time, after removal of sleep time [19, 20]. Blocks of 20 consecutive minutes of zero counts were considered as non-wear-time of the device and discarded from the analysis.

The investigation team verified that the data were complete using version 5.6 of the Actilife software (ActiGraph, Pensacola, FL). Data were collected at a sampling rate of 80 Hz, downloaded using 1 s epoch, which were subsequently aggregated for periods of 15 s [21]. The cut-points capture the sporadic nature of children’s activity and provide the best classification accuracy among the currently available cut-points for physical activity in children [20]. Specifically, sedentary time was defined as time accumulated at ≤ 25 activity counts/15 seconds, ≥ 26–573 activity counts/15 seconds for light physical activity, ≥ 574–1002 activity counts/15 seconds for moderate physical activity, ≥ 1003 activity counts/15 seconds for vigorous physical activity, and ≥ 574 activity counts/15 seconds for moderate-to-vigorous physical activity [21]. Time (min/day) spent sedentary, in light, and moderate-to-vigorous physical activity was calculated and used in analysis.

### Sociodemographic variables

A family demographic questionnaire was completed by the parents or legal guardians. Full details of the questionnaires are provided elsewhere [13, 14]. Parents were asked about the children’s sex, age, and race/ethnicity (white/caucasian, black, mixed, or other). Total annual family income was used as an indicator at the household level and was categorized into four categories based on data distribution. These categories represent increasing levels of annual income (Brazilian currency), so that those with the lowest income were organized in the first category and those with the highest income in the last: <R$19,620 (level 1); 19,621 to 32,700 (level 2); 32,701 to 58,860 (level 3) and > R$58,861 (level 4). The mother’s educational level was divided into three categories: incomplete high school, complete high school/incomplete higher education and complete higher education.

### Statistical analysis

The Kolmogorov-Smirnov test was used to assess data normality. Data is reported using means with 95% confidence interval (95%CI) and frequency as well as percentage (%) for categorical variables. For the comparison of categorical variables, we used the chi-square test and for continuous variables, we used the analysis of variance with one factor. Significant differences by nutritional status categories were analyzed by overlapping 95%CI, with a significant difference being considered when there was no overlap of the 95%CI; and no difference was considered when one of the 95%CI was partially included by the other [22].

Associations between variables were initially evaluated via Spearman correlation. Linear regression models were performed to estimate β-coefficient and 95%CI for the association between breastfeeding, parental BMI and birth weight (independent variables) with obesity indicators during late childhood (dependent variables). Separate models were determined for each independent variable adjusted for potential confounders mutually adjusted to each other. Model 1 was adjusted for school, sex, age, race/ethnicity and annual household income. Model 2 was adjusted for school, sex, age, race/ethnicity, annual household income, sedentary time and moderate-to-vigorous physical activity. The level of statistical significance was set as p < 0.05. Analyzes were performed using the Statistical Package for the Social Sciences V22 software (SPSS Inc., IBM Corp., Armonk, New York, NY, USA) [23].

## Results

There were no significant differences (p > 0.05) between the participants regarding parental BMI, birth weight and obesity indicators between those with complete data and those who did not answer the question on breastfeeding. The final sample size with complete data consisted of 402 (50.2% girls; mean age: 10.0 years [95%CI: 9.9; 10.1]) children. Sample characteristics are shown in Table 1 according to nutritional status categories. Based on nutritional status categories, most children were classified as eutrophic (58.2%) followed by overweight (20.9%) and obese (17.2%). Sex, age, race/ethnicity, annual family income, mother’s educational level, breastfeeding, sedentary time and light and moderate physical activity intensity were not statistically different between the nutritional status categories. There was, however, a significant difference between the nutritional status categories in terms of birth weight, parental BMI, WC and %BF (Table 1).

**Table 1:** Characterization [n (%) or mean (95%CI)] of the sample according to nutritional status

Variables	Underweight (n = 15)	Eutrophic (n = 234)	Overweight (n = 84)	Obese (n = 69)	p-value^1^
Sex [n (%)]					0.106
Boys	7 (46.7)	114 (48.7)	36 (42.9)	43 (62.3)	
Girls	8 (53.3)	120 (51.3)	48 (57.1)	26 (37.7)	
Age [years; mean (95%CI)]	10.0 (9.7; 10.3)	10.0 (9.9; 10.1)	10.0 (9.9; 10.1)	10.1 (9.9; 10.2)	0.748
Race/ethnicity [n (%)]					0.856
White/caucasian	13 (86.7)	172 (73.5)	63 (75.0)	56 (81.2)	
Black	0 (0.0)	15 (6.4)	4 (4.8)	7 (10.1)	
Mixed	2 (13.3)	35 (15.0)	12 (14.3)	4 (5.8)	
Others	0 (0.0)	12 (5.1)	5 (5.9)	2 (2.9)	
Annual family income [n (%)]					0.915
<R$ 19,620	6 (40.0)	78 (33.3)	27 (32.1)	20 (29.0)	
R$ 19,620–32,700	4 (26.7)	61 (26.1)	18 (21.4)	18 (26.1)	
R$ 32,701–58,860	2 (13.3)	56 (23.9)	23 (27.5)	17 (24.6)	
>R$ 58,860	3 (20.0)	39 (16.7)	16 (19.0)	14 (20.3)	
Mother’s educational level [n (%)]					0.382
Incomplete high school	10 (66.7)	156 (67.7)	54 (64.3)	51 (73.9)	
Complete high school/incomplete higher education	5 (33.3)	62 (26.5)	25 (29.7)	11 (15.9)	
Complete higher education	0 (0.0)	16 (6.8)	5 (6.0)	7 (10.1)	
Breastfeeding [month; mean (95%CI)]	6.21 (4.34; 8.08)	6.71 (6.28; 7.15)	6.53 (5.74; 7.31)	5.45 (4.55; 6.36)	0.086
Birth weight [g; mean (95%CI)]	2836.4 (2572.7; 3100.1)	3159.0 (3084.6; 3233.4)	3276.2 (3149.6; 3402.7)	3321.5 (3193.5; 3449.5)	< 0.001
Maternal BMI [kg/m^2^; mean (95%CI)]	23.8 (21.7; 26.0)	24.9 (24.3; 25.4)	27.6 (26.3; 29.2)	27.8 (26.4; 29.2)	< 0.001
Paternal BMI [kg/m^2^; mean (95%CI)]	25.3 (23.8; 26.8)	26.9 (26.2; 27.6)	28.9 (27.9; 29.9)	27.5 (26.4; 28.6)	< 0.001
Children BMI [kg/m^2^; mean (95%CI)]	13.5 (13.1; 13.8)	17.0 (16.8; 17.2)	21.5 (21.2; 21.7)	27.0 (26.1; 27.9)	< 0.001
Waist circumference [cm; mean (95%CI)]	54.2 (52.8; 55.6)	60.5 (59.9; 61.1)	71.3 (70.4; 72.3)	83.6 (81.4; 85.7)	< 0.001
Body fat [%; mean (95%CI)]	9.1 (7.1; 11.0)	17.8 (17,2; 28.4)	27.7 (26.7; 28.6)	36.2 (34.2; 38.1)	< 0.001
Accelerometry [min/day; mean (95%CI)]					
Sedentary time	490.9 (456.1; 525.7)	497.9 (488.8; 507.2)	503.2 (488.1; 518.2)	507.6 (487.4; 527.7)	0.716
Light physical activity	342.4 (322.1; 362.6)	336.6 (329.4; 343.8)	336.5 (323.3; 349.9)	332.8 (318.9; 346.6)	0.923
Moderate physical activity	46.4 (36.9; 55.8)	42.5 (40.1; 44.9)	38.4 (34.9; 41.8)	41.3 (37.6; 44.9)	0.210
Vigorous physical activity	19.9 (14.9; 25.0)	19.7 (17.9; 21.4)	15.0 (13.1; 16.9)	13.7 (11.2; 15.3)	< 0.001
Moderate-to-vigorous physical activity	66.2 (52.8; 79.9)	62.2 (58.3; 66.1)	53.4 (48.3; 58.5)	54.5 (49.1; 59.9)	0.023

R$: Brazilian currency; BMI: body mass index; ^1^Chi-square test or analysis of variance.

Table 2 presents the results of the correlation analysis describing associations between breastfeeding, parental BMI and birth weight with obesity indicators (BMI, %BF AND WC). Significant and positive correlations were observed between breastfeeding, maternal as well as paternal BMI and the children´s BMI, %BF and WC. Birth weight was weakly and positive associated with BMI and %BF but was not associated with WC (Table 2).

**Table 2:** Analysis of correlation of independent and dependent variables of children and parents

Variables	BMI (kg/m^2^)	Body fat (%)	WC (cm)	Breastfeeding (month)	Maternal BMI (kg/m^2^)	Paternal BMI (kg/m^2^)	Birth weight (g)
BMI (kg/m^2^)	1.00	0.921*	0.900*	-0.132*	0.320*	0.173*	0.165*
Body fat (%)	-----	1.00	0.834*	-0.112*	0.308*	0.198*	0.161*
WC (cm)	-----	-----	1.00	-0.097*	0.271*	0.153*	0.164
Breastfeeding (month)	-----	-----	-----	1.00	0.054	0.014	0.155*
Maternal BMI (kg/m^2^)	-----	-----	-----	-----	1.00	0.189*	0.095
Paternal BMI (kg/m^2^)	-----	-----	-----	-----	-----	1.00	0.109
Birth weight (g)	-----	-----	-----	-----	-----	-----	1.00

BMI: body mass index; WC: waist circumference.

*p < 0.05 in the Pearson correlation.

Table 3 shows the results of the multivariate linear regression analyses in which Model 1 included sex, age, race/ethnicity and annual household income as covariates and Model 2 additionally included sedentary time and moderate-to-vigorous physical activity. In Model 1, we found positive and significant associations of maternal and paternal BMI as well as birth weight with all obesity indicators (children’s BMI, %BF and WC). Furthermore, breastfeeding was positively associated with %BF. In Model 2, maternal BMI and birth weight were positively associated with all obesity indicators, independent of sex, age, race/ethnicity, annual household income, sedentary time and moderate-to-vigorous physical activity.

**Table 3:** Association (β; 95%CI) between breastfeeding, parents’ body mass index and birth weight and obesity indicators

Independent variables	β	95%CI	p-value		β	95%CI	p-value		β	95%CI	p-value
	Body mass index (kg/m^2^)				Body fat (%)				Waist circumference (cm)		
Model 1^a^											
Breastfeeding (month)	0.043	-0.007; 0.095	0.083		-0.104	-0.010; -0.198	0.030		0.085	-0.036; 0.203	0.149
Maternal BMI (kg/m^2^)	0.266	0.176; 0.366	< 0.001		0.512	0.310; 0.715	< 0.001		0.589	0.410; 0.772	< 0.001
Paternal BMI (kg/m^2^)	0.128	0.028: 0.263	0.031		0.339	0.109; 0.600	0.008		0.253	0.015; 0.535	0.060
Birth weight (g)	0.003	0.001; 0.005	< 0.001		0.002	0.001; 0.004	0.003		0.003	0.002; 0.005	< 0.001
Model 2 ^b^											
Breastfeeding (month)	-0.006	-0.200; 0.188	0.954		0.025	-0.380; 0.430	0.904		-0.06	-0.469; 0.457	0.979
Maternal BMI (kg/m^2^)	0.228	0.142; 0.314	< 0.001		0.484	0.297; 0.671	< 0.001		0.509	0.304; 0.715	< 0.001
Paternal BMI (kg/m^2^)	0.083	-0.028; 0.194	0.141		0.256	0.021; 0.491	0.033		0.183	-0-078; 0.445	0.168
Birth weight (g)	0.001	0.001; 0.002	< 0.001		0.002	0.001; 0.003	0.021		0.003	0.001; 0.005	< 0.001

^a^ Model 1 including school as random effect adjusted for school, sex, age, race/ethnicity and annual household income;

^b^ Model 2 including school as random effect was adjusted for school, sex, age, race/ethnicity, annual household income, sedentary time and moderate-to-vigorous physical activity.

## Discussion

Obesity is a chronic inflammatory disease with a complex etiology that is related to social, environmental, behavioral, biological and genetic factors [24]. Based on this, and knowing the importance of parents in this process, this study verified the positive association between parental BMI and birth weight with various obesity indicators during late childhood. Furthermore, breastfeeding was positively associated with %BF. After considering moderate-to-vigorous physical activity and sedentary time only maternal BMI remained remained a significant correlate of all obesity indicators. The main result showed positively association between maternal BMI and birth weight with all obesity indicators, independently of sex, age, race/ethnicity, annual household income, sedentary time and moderate-to-vigorous physical activity in the Model 2.

Breastfeeding has been increasingly studied in the context of preventing chronic non-communicable diseases during life, including the reduction in the risk of obesity [9, 25]. Given the influence of the perinatal environment on future weight status recommendations for the prevention of childhood obesity encourage longer duration of breastfeeding [26]. The present study found an inverse relationship between the number of months of breastfeeding and %BF (Model 1). Several studies also showed a possible dose-response relationship between the duration of breastfeeding and the risk of obesity [9, 27]. However, our study, despite showing a significant association %BF, did not find it with other obesity indicators. As described by Ma et al. [9] some studies that used only BMI as a measure for obesity also found no association between these variables, which may have underestimated the results. These inconsistencies reinforce the importance of using different indicators of obesity in future research to understand the factors associated with it. Possible mechanisms for protecting effects of breast milk against childhood obesity may also be related to nutritional and behavioral explanations. Breast milk has greater nutritional value and more balanced energy supply when compared to formula feeding, in addition to having more bioactive substances, such as leptin and ghrelin, which can influence the increase in body fat [27]. In addition, it is likely that the introduction of other more caloric foods reflects the early replacement of breast milk with other foods.

The role of parents against obesity in their children extends from the first years of life, with breastfeeding, to childhood and adolescence, by encouraging and promoting healthy habits. Parents have a strong influence on the level of physical activity and diet of their children, with children’s behavior and diet shaped according to the conditions they are exposed to [28]. The present study presented data that reinforces this concept, by showing significant associations between maternal BMI (Model 2) and all obesity indicators, independently of sedentary time and moderate-to-vigorous physical activity in children. Such findings can be explained by several reasons, from genetic predisposition to behavioral and exposure to common obesogenic factors (unhealthy diet) [29].

With regard to combating childhood obesity, these findings reinforce the importance of parental involvement in interventions targeting childhood obesity that should already start during pregnancy and in the first years of life and continue throughout childhood and adolescence. The entire family needs to be aware of and take responsibility for future health outcomes as parent’s influence type and availability of various foods. They may also control the use of electronic equipment as well as offering family programs [30].

Consistent with previous work [29, 31], a linear regression (Model 2) that included school, sex, age, race/ethnicity, annual household income, sedentary time and moderate-to-vigorous physical activity, showed a stronger association between children´s BMI and maternal BMI compared to paternal BMI (β = 0.484 compared with β = 0.256). Furthermore, maternal BMI was significantly associated with all obesity indicators and paternal BMI was significantly associated with only %BF. A systematic review [29] showed that it is premature to conclude that maternal and paternal weight statuses are equal risk factors for children´s obesity. According to the authors, another key factor to be measured is the timing of maternal and/or paternal overweight or obesity. It is probable that maternal obesity during some critical developmental period (e.g., pregnancy) might be more influential than maternal obesity during other periods [32, 33]. Therefore, more rigorous studies should be conducted to compare the relative impact of paternal and maternal weight on children’s overweight or obesity during dissimilar developmental periods of the parental and childhood life course.

The present study also presented data related to birth weight, and the direct association between birth weight with all obesity indicators (BMI, BF%, WC; Model 2). There is a large number of studies that indicate a higher risk for obesity in children who are born both overweight (> 4 kg) and those who are born under the ideal weight (< 2.5 kg) [34–36]. For instance, a meta-analysis of 66 studies from 26 countries demonstrated that high birth weight (> 4 kg) was positively associated with increased odds of childhood overweight (odds ratio: 1.66; 95%CI: 1.55; 1.77) compared to normal birth weight (< 2.5–4 kg) [36].

Some limitations of this study should be considered when interpreting the results. The cross-sectional design that does not allow establishing a cause-and-effect relationship; the non-representative sample; the city chosen for the study is not compatible with the reality of Brazil; information obtained from a recall on birth weight and breastfeeding duration increases the risk of recall bias; and the use of a questionnaire to obtain information about parents’ weight and height for the calculation of BMI can generate information bias; although the accelerometers have been shown to be a valid tool to measure activity of all levels of intensity [20, 21], the device cannot accurately capture various activities like upper body movements or cycling. The use of accelerometers to determine current activity levels in children along with the measurement of various obesity indicators, in addition to the quality of the procedures and methods in this study, on the other hand, are considerable strengths of this study.

Future studies, however, should consider more detailed information about pregnancy and breastfeeding, in addition to using more reliable instruments to obtain anthropometric values from parents. Furthermore, longitudinal studies that follow the evolution of growth and development of children with different durations of breastfeeding are needed. Finally, it should be remembered that the use of different obesity indicators must be considered.

## Conclusion

We showed positive associations of maternal BMI and birth weight with obesity indicators, independent of sex, age, race/ethnicity, annual household income, sedentary time and moderate-to-vigorous physical activity. The encouragement of healthy lifestyle behaviors for preventing and treating obesity in children and their parents, especially those with excess body weight, may be of importance to prevent cardiovascular disease. Public policies, therefore, should aim at decreasing the parental obesity levels and birth weight, in addition to considering the entire family unit in the treatment of childhood obesity from birth.

## Data Availability

The dataset used and analysed during the current study are available from the corresponding author on reasonable request.

## Abbreviations

BMI: body mass index.
ISCOLE: International Study of Childhood Obesity, Lifestyle and the Environment.
WC: waist circumference.
WHO: World Health Organization.
%BF: percentage of body fat.
95%CI: 95% confidence interval.
